# Efficacy of endometrial receptivity testing for recurrent implantation failure in patients with euploid embryo transfers: study protocol for a randomized controlled trial

**DOI:** 10.1186/s13063-024-08125-6

**Published:** 2024-05-28

**Authors:** Yao Lu, Xinyi Mao, Yaqiong He, Yuan Wang, Yun Sun

**Affiliations:** 1 Department of Reproductive Medicine, Ren Ji Hospital, Shanghai Jiao Tong University School of Medicine, Shanghai, 200135 China; 2 Shanghai Key Laboratory for Assisted Reproduction and Reproductive Genetics, Shanghai, 200135 China; 3 Shanghai Immune Therapy Institute, Shanghai Jiao Tong University School of Medicine-Affiliated Renji Hospital, Shanghai, China

**Keywords:** Endometrial receptivity testing, Recurrent implantation failure, Personalized embryo transfer, Randomized controlled trial, Live birth

## Abstract

**Background:**

Embryo implantation remains a critical barrier in assisted reproductive technologies. One of the main causes of unsuccessful embryo implantation is window of implantation (WOI) displacement, particularly in patients with recurrent implantation failure (RIF). Therefore, a reliable diagnostic tool for identifying the optimal WOI is essential. Previous data has suggested that a novel RNA-Seq-based endometrial receptivity testing (ERT) can diagnose WOI, guide personalized embryo transfer (pET), and improve pregnancy outcomes in patients with RIF compared to standard embryo transfer (sET). However, there is still a lack of evidence from randomized controlled trials (RCT) with sufficient power to determine whether pET based on ERT can increase the rate of live births as the primary outcome.

**Methods:**

This trial is a prospective, single-blind, parallel-group RCT (1:1 ratio of pET versus sET). Infertile women with RIF who intend to undergo frozen-thawed embryo transfer (FET) after preimplantation genetic testing for aneuploidy (PGT-A) with the availability of at least one euploid blastocyst for transfer will be enrolled and assigned into two parallel groups randomly. Participants in the intervention group will undergo ERT and then pET based on the results of ERT, while those in the control group will undergo sET. The primary outcome is live birth rate.

**Discussion:**

The findings of this study will provide evidence for the effect of pET guided by ERT on pregnancy outcomes in patients with RIF.

**Trial registration:**

Chinese Clinical Trial Registry ChiCTR2100049041. Registered on 20 July 2021.

**Supplementary Information:**

The online version contains supplementary material available at 10.1186/s13063-024-08125-6.

## Background

Infertility is regarded as a major public health concern, affecting 1 in 6 couples of reproductive age globally [1]. Since first introduced in 1978, in vitro fertilization (IVF) has brought hope and great benefits to millions of infertile couples. Currently, IVF is widely used all around the world, with more than 2,000,000 cycles being performed every year [2, 3].

Despite the substantial advancements in assisted reproductive technologies (ART), implantation remains a critical barrier in IVF [4]. More than 50% of IVF cycles still fail due to implantation failure [5]. Recurrent implantation failure (RIF) refers to the failure of implantation following repeated embryo transfer cycles, which is commonly encountered among IVF patients. RIF poses a significant challenge to clinicians and also causes great distress to infertile patients. Given the emotional and financial burden that comes with RIF, numerous interventions have been proposed to investigate and overcome this condition, while very few have proven effective [6].

Successful implantation requires a competent embryo, a receptive endometrium, and a synchronized dialog between the embryo and the endometrium [7]. Endometrial receptivity is a prerequisite for embryo implantation [8], and decreased endometrial receptivity accounts for up to two-thirds of implantation failure [9]. Endometrial receptivity occurs in a self-limited period during the mid-secretory phase, known as window of implantation (WOI). WOI typically falls between days 19 and 23 of the menstrual cycle [10] or on day 5 following progesterone treatment (P + 5) in a hormone replacement cycle [11]. However, the timing and duration of WOI are not identical for every woman. WOI displacement has been reported in about 25 to 50% of RIF patients and may be one of the main causes of implantation failure [12, 13]. Therefore, identification of the optimal WOI can help guide personalized embryo transfer (pET) and restore synchronicity between the embryo and the endometrium, which could potentially serve as a therapeutic approach to improve reproductive outcomes for patients with RIF.

Historically, Noyes et al. established histological criteria to “date” the endometrium [14], which has been used for decades, while being questioned regarding its accuracy, objectivity, and reproducibility [15, 16]. Despite the research that has been devoted to defining WOI through biomarkers and molecular indicators, reliable methods are still lacking in clinical practice [15, 17]. Therefore, valid and accurate methods are needed to identify the WOI and help determine the optimal timing for embryo transfer.

With the breakthroughs in high-throughput omics, transcriptomics has emerged as a promising diagnostic tool for endometrial receptivity. In 2011, a Spanish study first reported a microarray-based technique known as the endometrial receptivity array (ERA), which assesses endometrial receptivity status by analyzing the expression of 238 genes related to endometrial development [18]. Furthermore, in 2018, a research team from China established a novel endometrial receptivity testing (ERT) technology based on whole transcriptome RNA sequencing (RNA-Seq) analysis, combined with a machine learning algorithm, identifying 175 predictive genes [19]. Previous studies have shown that the analysis of gene transcriptomes, compared to histological methods, can more accurately and objectively assess endometrial receptivity and the timing of WOI with reproducible results [20–23].

Yet, the clinical efficacy of these new technologies requires further validation. Recently, there have been several randomized controlled trials (RCTs) evaluating the efficacy of ERA in the general population of IVF [24, 25], whereas no RCT has been conducted with regard to ERT and those with RIF. Notably, the ERT technology is gaining popularity due to the advantages of RNA-Seq, including high sensitivity, dynamic range, accurate quantification, and the ability to perform whole-transcriptome analysis [26]. Therefore, there is an urgent need for well-designed RCT to determine the efficacy of ERT, and whether pET based on ERT would improve pregnancy outcomes in women with RIF.

## Methods/design

### Study objective

This study aims to evaluate whether personalized embryo transfer (pET) based on ERT improves live birth rate compared with standard embryo transfer (sET) in patients with RIF.

### Design and setting

**Fig. 1:**
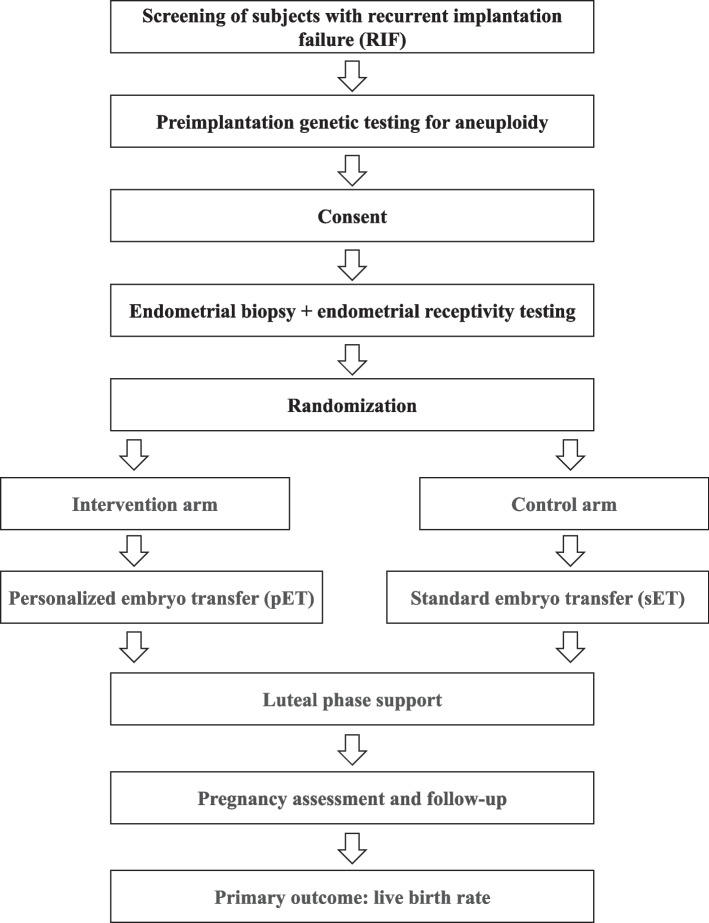
Flowchart of the study design

This is a prospective, single-blind, parallel-group RCT being performed in the Center for Reproductive Medicine, Ren Ji Hospital, Shanghai Jiao Tong University School of Medicine. The recruitment period will be from August 2021 to June 2024. All eligible patients will be randomly assigned to the intervention arm (pET) or control arm (sET) with a 1:1 ratio. The study design flowchart is shown in Fig. 1.

The study protocol was approved by the ethics committee of Ren Ji Hospital, Shanghai Jiao Tong University School of Medicine (reference number KY2021-081-B). The trial was registered in the Chinese Clinical Trial Registry (http://www.chictr.org.cn/; registration number ChiCTR2100049041). The trial will be conducted and reported in compliance with the Consolidated Standards of Reporting Trials (CONSORT) guidelines issued by the Enhancing Quality and Transparency of Health Research (EQUATOR) network. The study’s procedures will be fully explained to each participant by a research investigator. Each participant is required to sign an informed consent before taking part in the study.

### Eligibility criteria

#### Inclusion criteria

The inclusion criteria are as follows:

1. Women with RIF, which is defined as failure to achieve a clinical pregnancy under one of the following conditions:
  - Three or more embryo transfer cycles, with embryos transferred being of good quality (criteria of good-quality embryos are shown in Table 1)
  - Two or more euploid blastocyst transfer cycles
2. Women aged 20–39 years old at the time of autologous oocyte retrieval
3. Women who intend to undergo frozen-thawed blastocyst transfer (FET) after preimplantation genetic testing for aneuploidy (PGT-A)
4. Women who have at least one good-quality euploid blastocyst for transfer
5. Women who are capable of providing informed consent

**Table 1:** Criteria of good-quality embryo

Embryo	Scoring system	Criteria of good quality
Blastocyst (day 5 or day 6)	Gardner	≥ 4BC
Cleavage (day 3)	Puissant	7–10 cell 3; 4 or compact
	Peter	7–10 cell I; II or compact

#### Exclusion criteria

The exclusion criteria are as follows:

1. Women who have been diagnosed with diseases affecting the uterine cavity, including uterine malformation, submucous fibroids, intramural fibroids protruding into the uterine cavity, and untreated hydrosalpinx
2. Women or their partners with chromosomal abnormalities (not including chromosome polymorphisms)
3. Women with a history of recurrent pregnancy loss, defined as two or more failed pregnancies clinically recognized by ultrasonography or histopathologic examination
4. Women with thin endometrium (< 6 mm) before embryo transfer
5. Women with contraindications to endometrial biopsy, pregnancy, or assisted reproductive technology

### Sample size

Previous researches demonstrated that compared to sET, pET guided by ERT could increase pregnancy rates by about 25% in women with RIF [19, 27]. According to the retrospective data of our center, live birth among women with RIF following frozen-thawed euploid embryo transfer is approximately 35%. In the present study, we assume that the live birth rate will be improved from 35% in the sET arm to 60% in the pET arm.

For the sample size estimations, we will use a 2-tailed test with a statistical significance set at *α* = 0.05 and a statistical power of 1 − *β* = 0.80. The ratio between groups will be 1:1. Therefore, we need to include at least 118 women (59 women in each arm). Taking into consideration a dropout rate of 10%, we expect to include a total of 132 enrollees (66 enrollees in each arm).

### Randomization and blinding

All eligible women will be randomly assigned to one of two study arms according to a computer-generated dynamic block randomization sequence (sized 2, 4, and 6). The randomization sequence is generated by biostatisticians in the data coordinating center (DCC) using the SAS software version 9.2 (SAS Institute, Cary, NC) and has been input into an online randomization system by the DCC staff members prior to initiation of the study. The sequence will be kept strictly confidential by the DCC staff, thus to ensure that the study investigators are all blinded to the upcoming group allocation. On the day of endometrial biopsy, authorized investigators will log in to the password-protected account to get allocation information for eligible subjects. This is a single-blind clinical trial since it is not feasible to blind the doctors. Group allocation and ERT results will not be disclosed to all study participants.

### Screening

At the screening visit, all participants will be made aware of the trial and study plan. Eligible couples who are interested will sign a written informed consent and complete a comprehensive medical history review, physical examination, and pelvic ultrasound. The history of previous fertility treatments (including ovarian stimulation, oocyte retrievals, embryo transfers, hysteroscopy, and history of chronic endometritis) will be recorded. When necessary, screening for immunological and thrombophilic factors and chronic endometritis will be recommended.

**Fig. 2:**
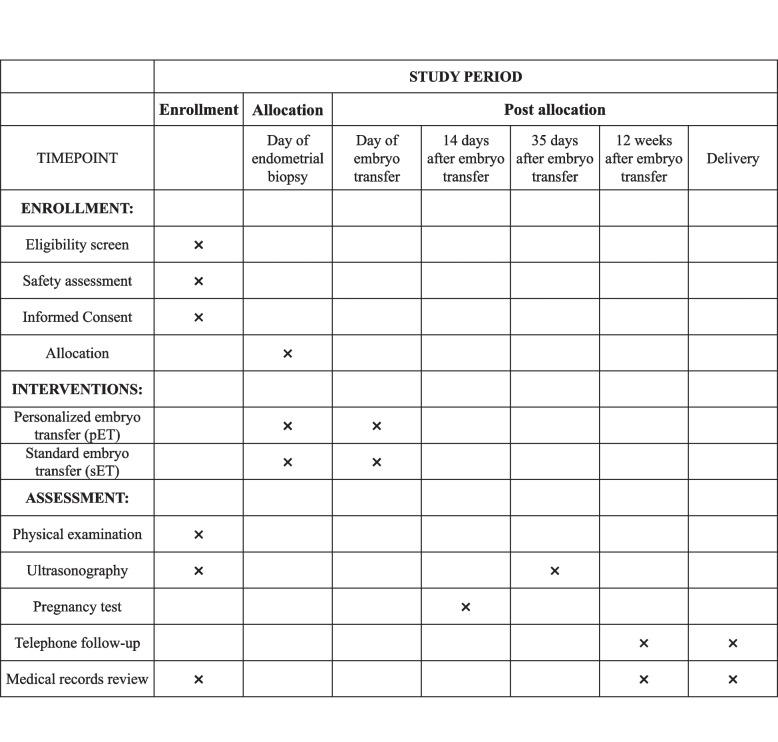
SPIRIT diagram for the schedule of enrollment, interventions, and assessments

Safety assessment includes but not limited to complete blood count (CBC), liver and renal function, fasting blood glucose, and blood pressure. Figure 2 (SPIRIT diagram) is a schedule of the study process including enrollment, interventions, and assessment. This protocol refers to the SPIRIT Reporting Guidelines [28], and the SPIRIT Checklist is shown in Additional file 1.

### Endometrial biopsy and endometrial receptivity testing

During the first menstrual cycle after enrollment, the endometrium will be prepared using hormone replacement treatment (HRT) to carry out ERT. Participants will be instructed to take oral estradiol valerate (Progynova, Delpharm Lille SAS) and/or estradiol tablets (Femoston, Abbott Biologicals B.V.) at a dose of 4 to 6 mg daily, starting from the 2nd to the 5th day of the menstrual cycle. The dose of estradiol will be individualized and can be increased to 8 mg daily if endometrial thickness is less than 7 mm after 14 days of administration. When the endometrial thickness is adequate, with serum progesterone level less than 1.5 ng/mL, vaginal progesterone gel (Crinone, Merck Serono) will be initiated within 24 h at a dose of 90 mg daily. Oral dydrogesterone (Duphaston, Abbott) will also be added 10 mg twice daily. The day of the first progesterone administration is defined as P + 0. An endometrial pipelle biopsy will be performed after five consecutive days (120 ± 6 h) of progesterone administration (P + 5). After endometrial pipelle biopsy, progesterone will be administrated for an additional 7 days and then discontinued.

The collected endometrial sample will be placed into 1.5 mL RNA-later buffer immediately for RNA stabilization, sealed, and cryopreserved at − 20 °C for preservation. Endometrial receptivity testing will be carried out within 7 days after sampling. The procedures will include total RNA extraction of endometrial sampling, RNA reverse transcription, library construction and sequencing, and data analysis using a machine learning algorithm. ERT results will be obtained within 15 days. Participants with non-informative results will be recommended to undergo a repeat testing. Any remaining specimens will be responsibly discarded after the endometrial receptivity analysis. No specimens will be retained for future use.

### Intervention

Participants who have a successful endometrial biopsy will be randomized into one of the two arms:

A) Intervention arm (pET arm): participants will undergo personalized embryo transfer based on ERT results. Specifically, the transfer will be performed at the standard timing (P + 5) if the ERT result is receptive. For participants with non-receptive results, relative to standard timing, the specific recommended adjustment ranged from 24 to 48 h later for pre-receptive results and 24 to 48 h earlier for post-receptive results, e.g., the transfer will be performed 24 h later (on P + 6) if the result shows 24 h pre-receptive.
B) Control arm (sET arm): participants will undergo standard embryo transfer on P + 5.

### Frozen-thawed embryo transfer

During the subsequent menstrual cycle, the same endometrial preparation regimen will be used as in the previous cycle. When the endometrial thickness is adequate, with serum progesterone level less than 1.5 ng/mL, the same regimen for luteal phase support (vaginal progesterone gel 90 mg daily and oral dydrogesterone 10 mg twice daily) will be added.

Participants in both arms will receive frozen-thawed single euploid blastocyst transfer. Serum human chorionic gonadotropin (hCG) levels will be measured 12–15 days after embryo transfer. If pregnancy is confirmed, luteal progesterone support will be continued until 10–12 weeks of gestation. All conceptions will be followed through the end of pregnancy.

### Discontinuation criteria

Reasons for dropping out of the trial are as follows:

1. Participants become pregnant spontaneously
2. Participants with no viable embryos for transfer after thawing
3. Participants who request to withdraw from the clinical trial

### Outcome and outcome assessments

#### Primary outcome

The primary outcome is live birth after frozen-thawed euploid blastocyst transfer, defined as the delivery of any newborns with signs of life at or beyond 28 weeks of gestation.

#### Secondary outcomes

The secondary outcomes include biochemical pregnancy, clinical pregnancy, implantation, ongoing pregnancy, and pregnancy loss. Biochemical pregnancy is defined as serum β-hCG level ≥ 10 mIU/mL measured 12–15 days after embryo transfer. Clinical pregnancy is defined as the detection of an intrauterine gestational sac via transvaginal ultrasound 35 days after embryo transfer. The implantation rate is calculated as the total number of intrauterine gestational sacs detected by transvaginal ultrasound divided by the total number of embryos transferred. Ongoing pregnancy is defined as a pregnancy beyond 12 weeks of gestation confirmed by a gestational sac with fetal heart activity using ultrasound.

#### Follow-up protocol

The first follow-up is at 12 weeks of gestation. Complications in the first trimester of pregnancy (including but not limited to spontaneous abortion, ectopic pregnancy, gestational trophoblastic disease, vaginal bleeding, or hyperemesis gravidarum) will be recorded by reviewing medical records or by telephone.

The second follow-up time point will be at delivery. The delivery information (including but not limited to gestational age, delivery mode, placenta abnormalities, or delivery complications) and neonatal information (including gender, birth weight, birth defects) will be obtained by standardized case report forms (CRFs) or by reviewing obstetric and neonatal medical records.

Participants will receive standard maternity care as per usual practice in addition to the study follow-up. Adverse events and concurrent drugs will be collected and documented at each visit throughout the trial. Participants who drop out or are lost to follow-up will be recorded.

### Adverse events

Adverse events (AEs) are defined as any untoward medical occurrences that arise during the trial period, whether or not they are thought to be related to the study intervention. Serious adverse events (SAEs) refer to any events that occur during the subject’s participation in this trial that meet one or more of the following criteria: death, life-threatening conditions, serious or persistent disability, necessitating inpatient hospitalization or extending an existing hospitalization, neonatal death up to 42 days after delivery, congenital abnormality or birth defect, or be otherwise deemed serious assessed by the principal investigator.

All AEs and SAEs will be thoroughly evaluated and recorded. Within 5 days, all SAEs will be reported to the principal investigator. Unexpected SAEs and SAEs that are possibly related to the study intervention should be reported within 24 h to the principal investigator. Upon receiving the report, the principal investigator will assess the seriousness and potential causality of the event in relation to the study intervention. Subsequently, any SAE will be reported to the ethics committee of Ren Ji Hospital, Shanghai Jiao Tong University School of Medicine, and DCC. Appropriate medical care and follow-up will be provided to the affected participant as per standard clinical practice. The principal investigator will maintain documentation of the SAE, including the initial report and any actions taken in response. If any SAE occurs and is thought to be related to the study intervention, 24 h unblinding will be available through the DCC for the individual participant if this is required by the principal investigator.

### Data management

Before the trial begins, all of the investigators, including physicians, nurses, and research assistants, will participate in a training course to ensure an understanding of the study protocol, the accuracy of outcome assessments, and the data collection. For each investigator, a protocol and standard operating procedures will be provided. All the research data will be collected using standardized CRFs where participants’ private information cannot be traced. The DCC is responsible for monitoring the trial. The DCC staff will routinely check the veracity, accuracy, and integrity of the data, to ensure the quality of the data collected.

### Data analysis plan

The data will be analyzed using SPSS (version 26.0, IBM Corp, Armonk, NY). Normally distributed continuous variables will be presented with mean ± standard deviation (SD), while non-normally distributed continuous variables with median and interquartile ranges (IQR). Independent samples *t*-tests or Wilcoxon’s rank sum test will be used to test the between-group differences as appropriate. Categorical variables will be presented with frequency and percentage. The Pearson chi-square and Fisher’s exact tests will be used to test the between-group differences as appropriate. A two-sided alpha level of 0.05 will be considered statistically significant.

The primary analysis will be conducted according to the intention-to-treat (ITT) principles. The primary outcome, live birth rate, will be compared between the two groups using the Pearson chi-square test. Women lost to follow-up will be considered as not having had a live birth. Secondary outcomes, including rates of biochemical pregnancy, clinical pregnancy, implantation, ongoing pregnancy, and pregnancy loss, will be analyzed using the Pearson chi-square test or Fisher’s exact test as appropriate. Secondarily, a per-protocol analysis will be performed among participants who complied with the trial protocol.

## Discussion

This is a trial aiming to evaluate the potential benefits of personalized embryo transfer, based on the endometrial receptivity testing, on live birth rate among patients with RIF undergoing FET after PGT-A. We plan to enroll 132 subjects from an academic reproductive center in China. The enrollment began in August 2021 and is expected to end in June 2024. The result of this RCT will provide high-quality evidence on the efficacy of ERT in predicting the WOI and improving pregnancy outcomes for patients with RIF. We assume that pET based on ERT will improve the live birth rate in patients with RIF.

RIF is a complex and challenging clinical condition in the field of ART. The failure of embryo implantation can be attributed to a variety of factors, including embryo quality, endometrial receptivity, or both [29, 30]. In the past few decades, many interventions have been proposed to improve embryo quality or endometrial receptivity, including techniques such as PGT-A to improve embryo selection, hysteroscopy to assess the uterine cavity, parental karyotype analysis to rule out chromosomal abnormalities, and screening for endocrine, immunological, and thrombophilia factors that may impact implantation [6, 30]. Additionally, synchronization between the endometrium and the embryo is another critical factor involved in successful implantation. Displacement of WOI has been suggested as one of the primary causes of failure for a euploid embryo to implant [31]. With the development of high-throughput omics, an increasing number of studies have focused on the discovery of gene expression related to endometrial receptivity [18, 20–22]. A novel endometrial receptivity testing technology based on RNA-Seq was thereby developed to uncover endometrial receptivity biomarkers through transcriptome analysis and to predict the ideal WOI [19].

However, clinical evidence regarding the effect of ERT remains limited. Only two clinical studies have been identified on this topic. He et al. conducted a prospective observational study including 142 women with RIF; results showed that WOI displacement was observed in 30.4% of cases, WOI narrowing was observed in 58.8% of cases, and pET based on ERT substantially increased the rate of intrauterine pregnancy (63.6% vs. 40.7%, *p* = 0.111) compared to that of sET when transferring blastocysts [19]. Additionally, Chen et al. reported similar findings in a retrospective study using propensity score matching, demonstrating that ERT-guided pET could significantly improve clinical pregnancy (50.0% vs. 16.7%, *p* = 0.001) and live birth rates (42.9% vs. 14.3%, *p* = 0.004) compared to pinopode-guided pET [23]. It should be noted that both studies were conducted in a non-randomized setting; thus, the efficacy of ERT needs to be further validated through well-designed randomized trials.

Thus, the purpose of the present study is to conduct a prospective, randomized, single-blind, clinical trial in patients with RIF and to evaluate the effect of ERT on live birth rate. Women with at least three unsuccessful good-quality embryo transfer cycles or two euploid embryo transfer cycles will be enrolled because we intend to include women who are more likely to have decreased endometrial receptivity and WOI displacement, so they can potentially benefit from ERT. To further minimize the confounding factors related to embryo quality, we have elected to include only participants who have undergone PGT-A treatment and have at least one good-quality euploid blastocysts for transfer. The results of this trial will provide high-quality evidence of the efficacy of ERT in the treatment of RIF.

Furthermore, it is also important for further research to consider the economic implications of ERT, including direct costs associated with the testing procedure, additional costs related to the testing cycle, and long-term economic implications of this intervention. Thorough cost-effectiveness analyses are necessary to inform decision-making regarding the adoption of ERT in clinical practice.

## Trial status

The study enrollment began in August 2021 and is expected to end in June 2024. The follow-up will continue and data collection will be completed in June 2025. At the time of the manuscript submission, the enrollment is ongoing, and more than 72 women have been recruited. The trial protocol is version 2.1, dated 25 March 2023.

### Supplementary Information

**Additional file 1.** SPIRIT 2013 Checklist: Recommended items to address in a clinical trial protocol and related documents.

**Additional file 2.** Model consent form.

## Data Availability

The datasets generated and processed during the current study are available from the corresponding author upon reasonable request.

## Abbreviations

AE: Adverse event
ART: Assisted reproductive technology
CBC: Complete blood count
ChiCTR: Chinese Clinical Trial Registry
CONSORT: Consolidated Standards of Reporting Trials
CRF: Case report form
DCC: Data Coordinating Center
ERA: Endometrial receptivity array
ERT: Endometrial receptivity testing
FET: Frozen embryo transfer
hCG: Human chorionic gonadotropin
HRT: Hormone replacement therapy
IQR: Interquartile ranges
ITT: Intention-to-treat
IVF: In vitro fertilization
pET: Personalized embryo transfer
PGT-A: Preimplantation genetic testing for aneuploidy
RCT: Randomized controlled trial
RIF: Recurrent implantation failure
RNA-Seq: RNA sequencing
SAE: Serious adverse event
SD: Standard deviation
sET: Standard embryo transfer
WOI: Window of implantation
