# Supplementary material for: Efficacy of endometrial receptivity testing for recurrent implantation failure in patients with euploid embryo transfers: study protocol for a randomized controlled trial
Source: Trials. 2024 May 28;25:348. doi: 10.1186/s13063-024-08125-6 (PMC11134766; doi:10.1186/s13063-024-08125-6)
Supplement: Supplementary file 2 — Additional file 2. Model consent form. [file 13063_2024_8125_MOESM2_ESM.docx]

**Informed Consent Form**

**Efficacy of the endometrial receptivity testing for recurrent implantation failure in patients with euploid embryo transfers**

Dear Patient:

We invite you to participate in the study entitled “Efficacy of the endometrial receptivity testing for recurrent implantation failure in patients with euploid embryo transfers”. This study will be conducted at the Reproductive Medicine Center of Ren Ji Hospital Affiliated to Shanghai Jiao Tong University School of Medicine, with an expected enrollment of 132 voluntary participants. This study has been reviewed and approved by the Ethics Committee of Ren Ji Hospital Affiliated to Shanghai Jiao Tong University School of Medicine.

This informed consent form will provide you with information to help you decide whether to participate in this study or not. Your participation in this study is entirely voluntary, and your decision will not affect your treatments, rights and benefits at our hospital. If you choose to participate in this study, our research team will make every effort to ensure your safety and rights during the process.

Please carefully read this informed consent form. If you have any questions, please consult the researcher explaining the informed consent form to you.

**I. Background Information**

In the 21st century, infertility has become the third most common disease in addition to tumor and cardiovascular disease, posing an unprecedented crisis to human fertility. Despite the substantial advancements in assisted reproductive technologies, embryo implantation remains a critical barrier, and its success largely depends on the interaction between the endometrium and the embryo. Recurrent implantation failure refers to the failure of implantation following repeated embryo transfers cycles, which is commonly encountered, poses significant challenges to clinicians and also causes great distress to patients.

There are three key factors for a successful embryo implantation: a competent embryo, a receptive endometrium and a synchronized dialogue between the embryo and the endometrium. Successful pregnancy can only be achieved when all three conditions are met simultaneously. Many studies have shown that among the reasons for recurrent implantation failure, embryo factors account for only one-third, while two-thirds of the reasons are due to inadequate endometrial receptivity.

Currently, the assessment of endometrial receptivity mainly relies on ultrasound monitoring of endometrial thickness and its appearance. However, ultrasound observation is dependent on subjective judgment by the examiner, and studies have found that the window of implantation assessed by ultrasound evaluation is not completely consistent with the histological level. Endometrial receptivity testing could potentially detect endometrial receptivity more accurately. Preliminary data has shown that about 40% of those with recurrent implantation failure has shifting of the window of implantation, thus personalized embryo transfer based on endometrial receptivity testing results may improve pregnancy outcomes. Yet, clinical benefits of endometrial receptivity testing remain uncertain.

**II. Research Objectives**

To evaluate whether personalized embryo transfer based on endometrial receptivity testing improves pregnancy outcomes in patients with recurrent implantation failure compared to conventional embryo transfer.

**III. Trial Subjects**

Patients with recurrent implantation failure, with an expected enrollment of 132 voluntary participants.

Inclusion criteria:

1. Women with recurrent implantation failure, which is defined as failure to achieve a clinical pregnancy under one of the following conditions:
   1. Three or more embryo transfer cycles, with embryos transferred being of good quality;
   2. Two or more pre-implantation genetic tested embryo transfer cycles;
2. Women aged 20-39 years old at the time of oocyte retrieval;
3. Women who intend to undergo frozen-thawed embryo transfer after preimplantation genetic testing for abnormal embryos;
4. Women who have at least one good-quality normal blastocyst for transfer;
5. Women who are capable of providing informed consent.

Exclusion criteria:

1. Women who have been diagnosed with diseases affecting the uterine cavity, such as uterine malformation, submucous fibroids, intramural fibroids protruding into the uterine cavity and untreated hydrosalpinx (diseased fallopian tubes);
2. Women or their partner with chromosomal abnormalities;
3. Women with a history of recurrent pregnancy loss, defined as two or more failed pregnancies recognized by ultrasound or removed pregnancy tissue;
4. Women with thin endometrium (< 6 mm) before embryo transfer;
5. Women with an inability to undergo endometrial biopsy, pregnancy or assisted reproductive technology.

**IV. Research Procedure**

If you are enrolled in this study, during the first menstrual cycle after enrollment, you will receive a mock cycle to carry out endometrial biopsy for endometrial receptivity testing. The endometrium will be prepared using standard hormone replacement treatment. An endometrial biopsy will be performed after 5 days of progesterone administration. endometrial receptivity testing will be carried out and results will be obtained within 15 days.

After endometrial biopsy, you will be randomly assigned to one of two study groups: the intervention group or the control group. Your chance of being assigned to either group is the same. If assigned to the intervention group, you will undergo personalized embryo transfer based on endometrial receptivity testing results. If allocated to the control group, a standard embryo transfer will be performed. Group placement and endometrial receptivity testing results will be disclosed only to your attending, while you will not be informed until you finish a subsequent embryo transfer cycle. All remaining endometrial tissue after endometrial receptivity testing will be discarded according to our regulations for biological samples.

If you agree to participate in this study, the biopsy cycle and the embryo transfer cycle will take approximately 2-3 months. If pregnant, follow-up will continue until delivery, and information during your pregnancy and delivery will be collected. All personal information collected during this study will be stored by the research team at the Reproductive Center of Ren Ji Hospital. The research team will ensure the confidentiality of your personal information. You can choose to withdraw from the study at any time without losing any benefits you are entitled to receive.

**V. Alternative Treatments**

Despite various therapeutic interventions have been proposed to overcome recurrent implantation failure, very few are evidence based in clinical practice. Participating in this study may or may not improve your pregnancy success rate. You can choose to:

1. Do not participate in this study and continue your routine treatment.
2. Participate in other studies.
3. Give up assisted reproductive treatments.

Please consult with your doctor regarding your decision.

**VI. Possible Risks and Discomforts**

Endometrial biopsy for the endometrial receptivity testing will be collected concurrently with endometrial scratching, which is a routine clinical procedure that potentially benefits patients with recurrent implantation failure, and does not increase additional risks. The procedure is considered minimally invasive and generally takes 5-10 minutes. You may experience some discomfort, such as mild abdominal pain and vaginal bleeding during and after the procedure. If the first biopsy sample yields insufficient tissue, which affects result analysis, you may need to undergo sampling for endometrial receptivity testing again.

**VII. Expected Benefits**

Participating in this study may not directly benefit you. If you are assigned to the intervention group, you will receive endometrial receptivity testing based on endometrial receptivity testing results, potentially leading to a higher chance of embryo implantation rate. Additionally, this study may provide new information that could benefit other patients.

**VIII. Free Treatment**

You will not be required to bear any additional costs other than routine clinical checks and treatment if you participate in this study. Endometrial receptivity testing will be provided free of charge in this study.

**IX. Compensation**

The costs of frozen embryo transfer cycles during this study will be borne by the patients themselves. Doctors will make every effort to prevent and treat any damage that may result from this study. If any adverse events or injuries related to this study occur, appropriate treatment will be provided, and the corresponding diagnostic and treatment costs will be borne by the researchers. No financial compensation will be provided in this study.

**X. Indemnification**

If a participant suffers injury or damage due to the content of the study, appropriate treatment will be provided to eliminate harm to the patient, and in accordance with relevant laws and regulations, the corresponding treatment costs will be borne. Accidents caused by pre-existing conditions, as well as new diseases or exacerbation of diseases unrelated to this clinical study, are not included, but priority treatment can be provided at our hospital.

**XI. Confidentiality**

All your medical information will be kept confidential at all times. Only the research doctor will retain the participant's basic information, and code will be used to handle it in other project documents. Any public reports regarding the results of this study will not disclose your personal identity information. We will make every effort to protect the privacy of your personal medical data within the legal limits.

In necessary circumstances, researchers, research management authorities, ethics committees, and superior verification departments will be allowed to access your medical records and related information under the premise of signing a confidentiality agreement. When you sign this informed consent form, it signifies your agreement to the use of your personal and medical information for the purposes described above.

**XII. Voluntariness**

You have the option to choose not to participate in this study, or you may inform the researchers at any time to withdraw from the study. Your data will not be included in the study results, and your medical treatment and rights will not be affected by this decision. If you require other treatments, or if you fail to adhere to the study protocol, or if you experience any injuries related to the study or any other reasons, the research physician may terminate your participation in the study. You are entitled to be informed about information and progress related to this study at any time, and if new safety-related information related to this study arises, we will promptly notify you.

**XIII. Participant Responsibilities**

As a research participant, you have the following responsibilities: providing truthful information about your medical history and current physical condition; informing the research doctor of any discomfort or all concomitant medications during this study period; informing the research doctor if you have recently participated in other studies or are currently participating in other studies.

**XIV. Contact Information**

If you have any questions related to this study, or if you experience any discomfort or injuries during the study, or if you have any questions regarding the rights of participants in this study, you can contact Dr. Lu at 18918358590.

If you have any questions or complaints about the researchers during the study, you can contact the Ethics Committee of Ren Ji Hospital affiliated to Shanghai Jiao Tong University School of Medicine, at the following telephone number: 021-68383364.

**Participant’s Agreement:**

I have read the information provided above. I have asked all the questions I have at this time. I voluntarily agree to participate in this study.

Signature of Research Participant:

Date:

Signature of Research Team Member Obtaining Consent:

Date:
